# Melt Electrospinning of PET and Composite PET-Aerogel Fibers: An Experimental and Modeling Study

**DOI:** 10.3390/ma14164699

**Published:** 2021-08-20

**Authors:** Lasse Christiansen, Leonid Gurevich, Deyong Wang, Peter Fojan

**Affiliations:** Department of Materials and Production, Aalborg University, 9220 Aalborg, Denmark; lch@ucn.dk (L.C.); lg@mp.aau.dk (L.G.); dw@mp.aau.dk (D.W.)

**Keywords:** electrospinning, porous materials, composite fibers, thermal insulation, finite element modeling, logistic viscosity model

## Abstract

Increasingly advanced applications of polymer fibers are driving the demand for new, high-performance fiber types. One way to produce polymer fibers is by electrospinning from polymer solutions and melts. Polymer melt electrospinning produces fibers with small diameters through solvent-free processing and has applications within different fields, ranging from textile and construction, to the biotech and pharmaceutical industries. Modeling of the electrospinning process has been mainly limited to simulations of geometry-dependent electric field distributions. The associated large change in viscosity upon fiber formation and elongation is a key issue governing the electrospinning process, apart from other environmental factors. This paper investigates the melt electrospinning of aerogel-containing fibers and proposes a logistic viscosity model approach with parametric ramping in a finite element method (FEM) simulation. The formation of melt electrospun fibers is studied with regard to the spinning temperature and the distance to the collector. The formation of PET-Aerogel composite fibers by pneumatic transport is demonstrated, and the critical parameter is found to be the temperature of the gas phase. The experimental results form the basis for the electrospinning model, which is shown to reproduce the trend for the fiber diameter, both for polymer as well as polymer-aerogel composites.

## 1. Introduction

Polymer micro- and nanofibers can be created in several ways, and one fast and efficient process is electrospinning [1]. Electrospinning occurs when a droplet of fiber-forming solution or melt is placed in an electric field. As the field strength surpasses the surface tension, a Taylor cone emerges and a fiber emanates from the droplet [2]. The critical point of melt electrospinning occurs when the temperature of the melt is in a range above or equal to the glass transition temperature, but below the decomposition temperature [2]. The upper temperature limit of melt electrospinning is actually lower than the decomposition temperature for the polymer due to oxidation and the viscosity of the polymer melt. The process can be controlled through many parameters [3,4,5]. In the past, several efforts to electrospin advanced composite materials [6,7,8], and to upscale the process to an industrial scale [9,10], have been made. Among these types of advanced materials [8,11,12,13] are aerogel/polymer composite fibers, described earlier by this group [14,15]. Such materials allow for a combination of the incredible thermal insulation properties of silica-based aerogel and the mechanical strength and ease of handling of polymer fibers. So far, these fibers have been produced with organic solvents that are environmentally hazardous [16,17,18] and can compromise the properties of the aerogel [19]. Solvent-free production of aerogel composite fibers can enable their application in a whole range of new fields, including building insulation [20], high-performance clothing [21], aerospace suits [22], and the insulation of batteries in electric vehicles [23].

Several approaches to the simulation of the electrospinning process have been formulated over the past years, including numeric motion simulations [24] and crystallization simulations [25]. These approaches cover various electrospinning scenarios, including centrifugal electrospinning [26], morphology simulations [27], and fiber diameter simulations [25] in numerical environments. Furthermore, the finite element method (FEM) has been used for field calculations [28] and for the calculation of the mechanical properties of the resulting fibers [29,30]. However, the simulation of the actual fiber formation by FEM is not covered in the literature. The finite element method is versatile as it can simulate multiphysics problems [31] as well as integrate with digital design and manufacturing environments [32]. With the increasing robustness of computational fluid dynamics algorithms, this method can also be implemented for the electrospinning process. This has been shown for the electrospinning of liquids with a constant viscosity [31]. However, this approach is not suitable for modeling melt electrospinning since it involves large variations in viscosity during the glass transition of the fiber formation process. The introduction of a parametric dependence can address this problem. 

In the solvent-free melt electrospinning process, the main parameter is the temperature-dependent viscosity, which increases drastically upon cooling of the melt [2,33]. Modeling based on a phenomenological temperature dependence can be used to evaluate the influence of other parameters on electrospun fiber morphology.

The present study reports a novel method for pneumatic transport of particulate matter into the core of melt electrospun fibers. Up until now, this has only been demonstrated for solvent electrospinning [14,15]. Melt electrospinning, on the other hand, offers solvent- free fiber formation, allowing the incorporation of dry porous particles without residual solvent, and avoiding their collapse due to solvent wetting. Specifically, polyethylene terephthalate (PET) and cellulose acetate butyrate polymer fibers were melt electrospun at various temperatures. Insugel™ aerogel particles were transported pneumatically into the core of the PET polymer fibers at different flow rates to create PET-Aerogel composites. These experiments formed the basis of the simulation of the fiber diameter with a viscosity ramping approach implemented as a temperature-dependent logistic viscosity model. 

## 2. Materials and Methods

**Figure 1:**
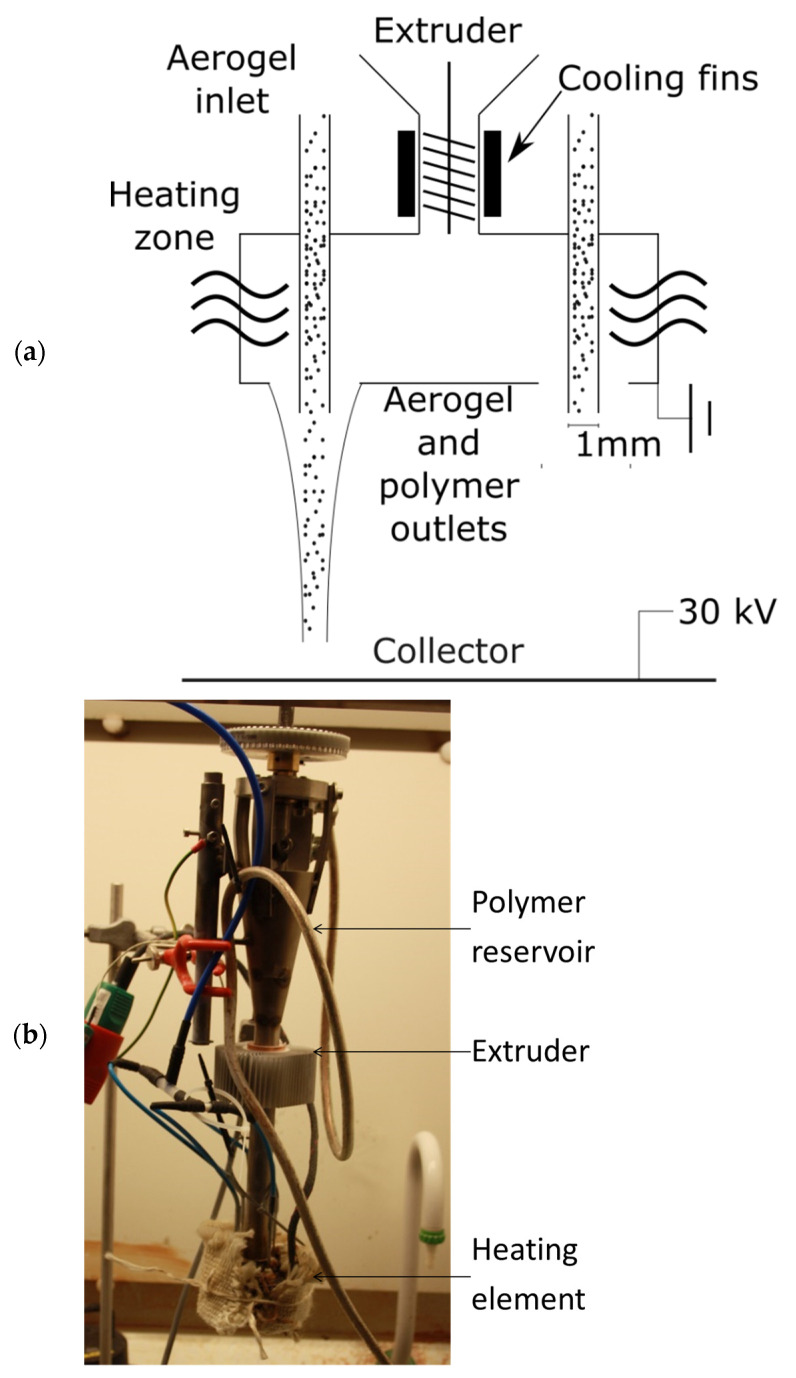
(**a**) Sketch and (**b**) image of the electrospinning setup.

PET (ES306313) was purchased from Goodfellow (UK), and cellulose acetate butyrate (Mw 30,000 g/mol) from Sigma Aldrich (Denmark); Insulgel™ hydrophobic silica aerogel particles with grain sizes in the range of 1–44 µm were purchased from Insulgel High-Tech (Beijing) Co., Ltd. (Beijing, China). The experiments were performed using a downward electrospinning setup, as shown in Figure 1, where a positive potential was applied to the collector while the spinneret was connected to ground. The electrospinning nozzles and aerogel outlets were 3 mm and 1 mm in diameter, respectively. The aerogel was transported into the spinneret by compressed dry air. 

The fibers were characterized by scanning electron microscopy (SEM, Zeiss XB40, Germany); the fiber diameter distribution was extracted from the SEM images. The presence of aerogel in the composite fibers was validated using energy dispersive X-ray spectroscopy (EDX, Thermo Scientific NORAN System 7, USA). 

### 2.1. Electrospinning Experiments

The electrospinning experiments consisted of a series of parametric variations used to find suitable settings for the production of aerogel-containing polymer fibers. The variation in temperature and distance formed the basis for the simulation work. 

First, PET and cellulose acetate butyrate were spun into fibers in order to investigate their spinnability. On the basis of these experiments, PET was chosen for further experiments as the equipment’s available temperature range fit this polymer better. 

In the second phase, the temperature and spinning distance were varied. The distance was varied between 7.4 and 21 cm, while the temperature was set to 280, 290, 300, and 320 °C. These experiments yielded an optimal spinning parameter set of 7.4 cm and 300 °C, which was used for the last production of polymer/aerogel composite fibers. 

While producing the aerogel-containing fibers, airflow was varied between 0.3 and 0.5 L/min in order to investigate the effect on fiber thickness and aerogel transport. This airflow was applied through the aerogel inlet and transported the aerogel particles into the middle of the electrospinning process. 

### 2.2. Multiphysics Simulations

The electrospinning process simulation was performed in three steps: (i) the electrospinning of polymer fibers without aerogel was simulated; (ii) a thermal simulation of the aerogel transport airflow was performed to obtain an average air temperature at the outlet; (iii) the average air temperature was used for the input parameters for the composite electrospinning simulation. 

During the first phase of simulations, the electrospinning process simulation relied on an FEM ramping approach in COMSOL Multiphysics 5.4, where a liquid with no temperature dependence on viscosity in a force field was simulated. The multiphysics consisted of heat transfer and laminar flow in a two-phase field. The liquid was injected into a domain with an applied body force, and the propagation of the liquid was simulated over a time span of 2 s in steps of 0.1 s. The liquid phase diameter at the opposite boundary was evaluated as a function of the applied force (corresponding to spinning distance) and temperature (corresponding to a higher cooling rate in the composite experiments). 

**Figure 2:**
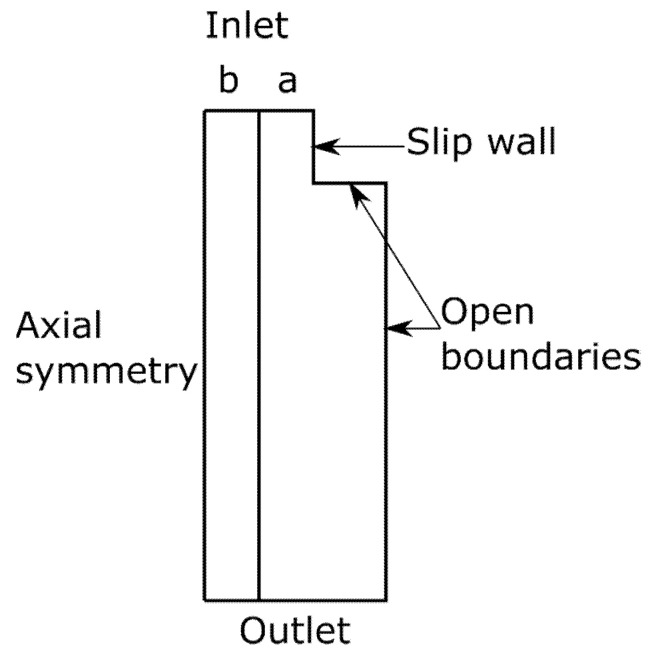
Simulation geometry and boundary conditions of the spinning process. Melted polymer is supplied through inlets a and b. The temperature at inlet a is set to either the heating mantle temperature or the airflow temperature from the thermal air transport simulation. Inlet b is set to the heating mantle temperature. Open boundaries allow air to enter or exit the model.

The simulations were carried out in a 2D axio-symmetric geometry (COMSOL Multiphysics 5.4), where two coupling terms were used to couple a two-phase flow with heat transfer in fluids. Figure 2 shows the simulation geometry. The inlet and outlet conveyed the molten polymer, the walls were set as an open boundary (to allow for airflow through the model) and a slip wall boundary condition. The model included heat transfer and a laminar two-phase flow with the applied body force due to the electrostatic force acting on the liquid. These were coupled so they would update material properties dependent on each other (thermal properties and viscosity). The coupling took place so that the thermal settings were resolved for a given time step, and the flow solver improved the temperature distribution. Subsequently, at the beginning of the next time step, the flow settings were imported by the thermal solver, ensuring an update of the material properties in a given region at all times. 

The solution was configured in two steps, a phase initiation step and a time-dependent step. The viscosity difference between the glass transition viscosity and the melt viscosity was ramped between 1 to 105 in steps of 10 by setting the initial phases equal to the previous solution and applying the new, higher glass transition viscosity. 

The dynamic viscosity, $\mu_{dynamic},$ was modeled as a logistic function. This approach has previously been used to describe the flow properties of other viscous liquids, such as blood [34] and asphalt [35,36], in other modeling systems. While the viscosity of the polymer melt is different from blood and asphalt, all have a strong viscosity dependence on environmental conditions that can be approximated with the same mathematical approach. This approximation can be implemented within the finite element environment, as it is easily tunable and fully differentiable and, at the same time, has been proven to describe a liquid that flows and eventually solidifies. This implementation allows for a parametric ramping approach, where the factor determines the order of magnitude of viscosity change from the melting temperature to the glass transition temperature:

(1) $$\mu_{dynamic}=\mu_{melt}+\frac{\mu_{glass}}{1+10^{\;\frac{2\cdot a}{T_{melt}-T_{glass}}\;\cdot \left(T-T_{melt}+\frac{T_{melt}-T_{glass}}{2}\right)}},$$

where $\mu_{dynamic}$ is the dynamic viscosity of the molten polymer, $\mu_{melt}$ is the viscosity at the melting temperature, $\mu_{glass}$ is the viscosity at the glass transition temperature, $T_{melt}$ is the melting temperature, $T_{glass}$ is the glass transition temperature, T is the temperature of the given element, and *a* is a number added to let a controlled fraction of the viscosity change happen between the glass transition temperature and the melt temperature. It is noted that the viscosity remains constant if *a* = 0.

The thermal conductivity, heat capacity, and density of the mixture in a given element were evaluated through a linear mixture model, where

(2) $$c_{p,tot}=c_{p,polymer}\cdot {\%}_{vol,polymer}+c_{p,air}\cdot {\%}_{vol,air},$$

(3) $$\lambda_{tot}=\lambda_{polymer}\cdot {\%}_{vol,polymer}+\lambda_{air}\cdot {\%}_{vol,air},$$

(4) $$\rho_{tot}=\rho_{polymer}\cdot {\%}_{vol,polymer}+\rho_{air}\cdot {\%}_{vol,air},$$

where $c_{p}$ is the heat capacity, λ is the thermal conductivity, ρ is the density, and ${\%}_{vol}$ refers to the volume percentage in a given element.

The pulling force on the droplet was applied to the elements within the finite element method. This was implemented as a description of Coulomb’s law,

(5) $$F\left(r\right)=qE\left(r\right)=-q\frac{\varphi }{r},$$

where *F* is the force, *q* is the charge density, *E* is the electrostatic field, φ  the electrostatic potential, and *r* the distance from the charged object. This is implemented as a body force acting on the volume element, so

(6) $$F\propto \frac{\rho }{d},$$

where *F* is the force on the volume element, ρ is the density, and *d* is the distance from the spinneret, as the charge and mass density are proportional. 

Since (1) the viscosity of suspensions is proportional to particle content, (2) the particle content in this study is low, and (3) the viscosity develops exponentially with temperature, it can be assumed that the temperature effect is dominant ($∆\mu_{T}>>∆\mu_{particle}$) [37,38]. Thus, the presence of aerogel particles is considered to be an inhomogeneity and does not affect the simulated properties [39]. 

In order to perform the parametric ramping, the electrospinning process can be described as a set of simulations with increased polymer viscosity. This was performed as six series of experiments, where the first experiment had a viscosity setting of $\mu_{glass}=\mu_{melt}$. For the next step, $\mu_{glass}$ was increased by a factor of ten. At the same time, the solution from the previous time step was used in the initial phase and temperature distribution so that the result would be updated to the new polymer properties. 

The parameters applied to this polymer flow model are shown in Table 1.

**Figure 3:**
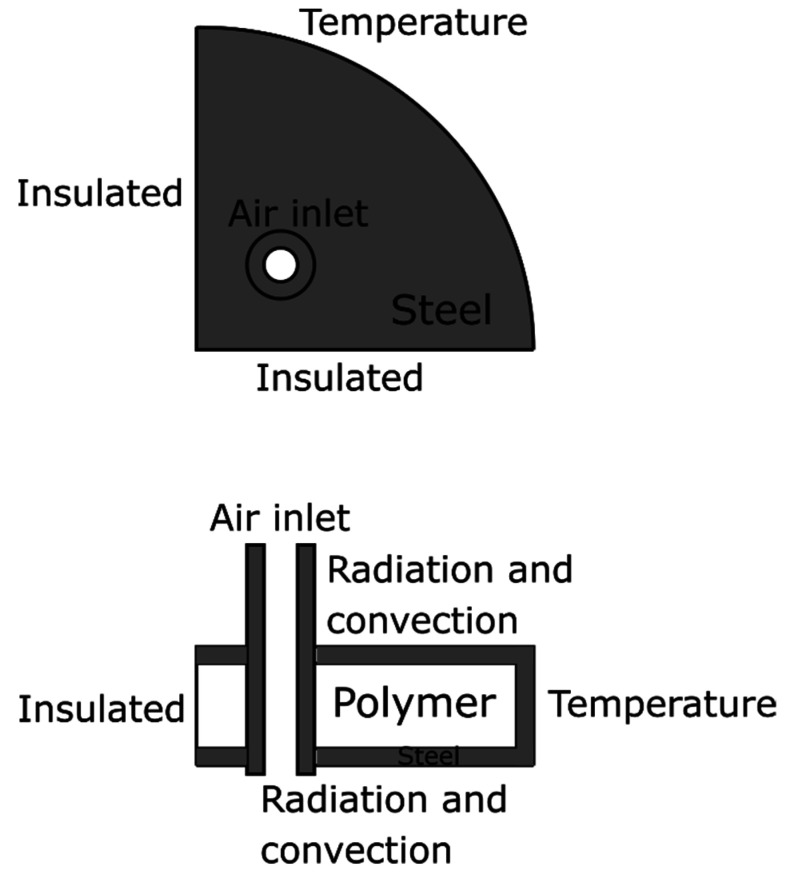
Subsection of the melt spinneret geometry and the boundary conditions. Airflow with room temperature was applied through the inner tube, and 300 °C was applied at the outer part. The casing and the tube were set as steel and the inner part as PET polymer.

A finite element model with temperature-flow coupling was used to estimate the temperature of the air and aerogel in the composite experiments. Figure 3 shows how thermal simulation was configured with geometry and boundary conditions. The flow was simulated according to the experiment, and the average output temperature was evaluated. This simulation was performed by heating the moving air, and the output was the average air temperature from the aerogel pipe. 

The simulations of the spinning with aerogel were performed as described for the general spinning, but with a lower input temperature in the fiber core. This corresponds to the area (a) in Figure 2.

## 3. Results and Discussion

**Figure 4:**
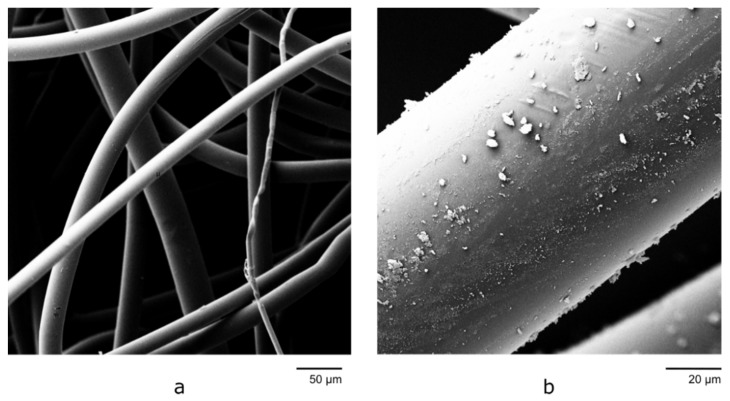
Electrospun PET fibers (**a**) without and (**b**) with aerogel.

Fibers were produced from both PET and cellulose acetate butyrate, yielding diameters in the micrometer range. Figure 4 shows representative images of polymer fibers Figure 4a), and composite fibers (Figure 4b). The two polymer types were both spinnable, but cellulose acetate butyrate showed thermal degradation at all temperatures with spinnable viscosity (150 °C and above). The PET fibers had small mean diameters and a stable electrospinning rate at 300 °C and a 7.4 cm spinning distance. Hence, these were chosen as the spinning parameters for further pneumatic experiments. All fibers had small, uniform diameters at high temperatures and smaller distances, but both the diameter and the variance increased with greater distances and lower temperatures. All spinning was stable at the chosen distance, but at greater distances, it tended to destabilize. The upper and lower distances mark the maximal and minimal spinning distance, respectively, where stable spinning could be maintained. Further details are shown in Table 2. 

**Figure 5:**
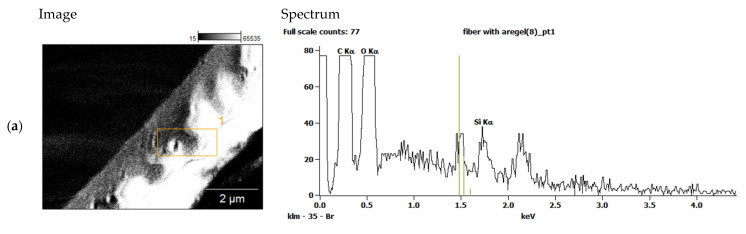

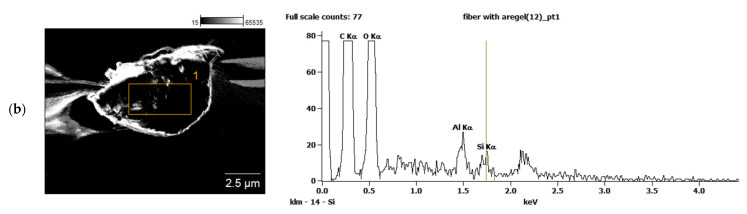
EDX spectra measured (**a**) on the surface and (**b**) in the cross-section of composite fibers. Both spectra show the presence of silicon, confirming the incorporation of aerogel particles into electrospun PET fibers.

The pure PET fibers exhibited a smooth surface, while the aerogel exhibited small particles on the composite fibers’ surface. This was also confirmed by EDX measurements of an aerogel-containing fiber, proving that the silica aerogel particles are both present on the surface and inside the fiber (Figure 5). This suggests that the aerogel particles are intermixed well with the polymer and were encapsulated in a polymer matrix within the fiber. It should be noted that unstable spinning at high airflow rates and low aerogel incorporation at low airflow rates place some limitations on the aerogel content of the fibers in the experimental setup used.

**Figure 6:**
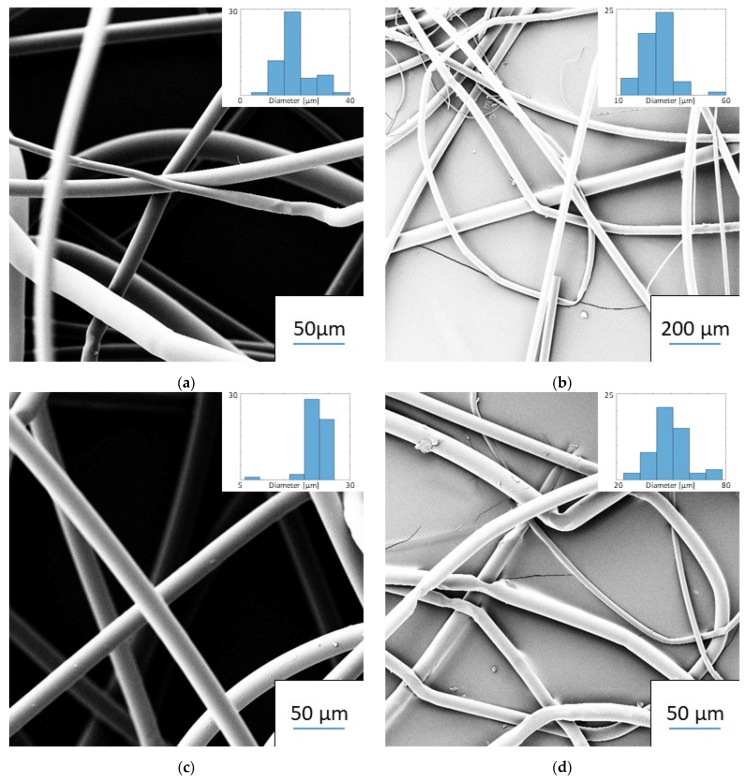
Images of PET fibers spun at 300 °C at different spinning distances: (**a**) 7.4 cm, (**b**) 10.1 cm, (**c**) 10.9 cm, and (**d**) 16.9 cm.

The mean size of the produced fibers without added aerogel varied between 12.4 and 91.2 µm for PET fibers, and 22.2 m and 39.0 µm for cellulose acetate butyrate fibers, depending on distance and temperature. Table 2 shows the average fiber diameters and standard deviations for all the experimental series for pure polymer fiber spinning. The table shows that spinning at a lower distance produced thinner fibers due to a higher electrostatic pulling force. Furthermore, increasing temperature also led to a decrease in the fiber diameters for both materials due to a reduction in viscosity. Figure 6 shows the fibers and their size distribution for all samples spun at 300 °C. This temperature and the distance of 7.4 cm were chosen for the spinning of aerogel-containing fibers, as they produced thin fibers with a narrow size distribution. The electrospinning at 320 °C produced thinner and more uniform fibers but did show signs of thermal degradation (fiber discoloration). Electrospinning of PET at temperatures below 280 °C was not possible due to high viscosity. Therefore, the spinnable range of non-degraded PET polymer fibers is between 280 and 300 °C. 

**Figure 7:**
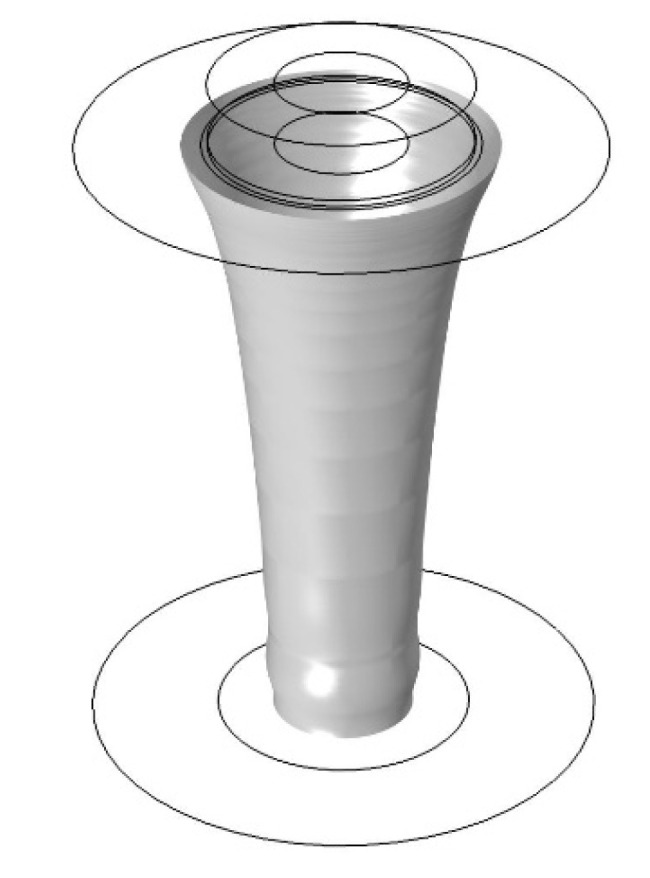
A 3D image of the polymer–air interface in the electrospinning simulation. The polymer is fed through the top at a defined temperature, and travels toward the outlet at the bottom.

**Figure 8:**
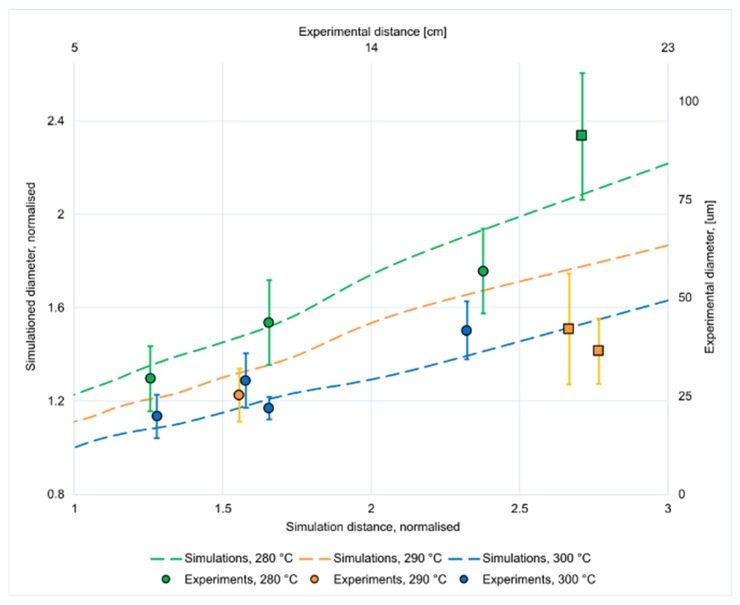
Simulated (lines) and measured (points with error bars) diameters for electrospun fibers at different distances and temperatures. Simulations are normalized and square data points mark unstable spinning conditions.

When the melt without temperature dependence was simulated in the initial simulation, a polymer jet was obtained. Upon introduction of temperature dependence and ramping up the proportionality factor *a*, the jet was used as an initial estimate for the next iteration. This was repeated as the proportionality factor was ramped between 1 and 10^5^. The fiber diameter at the outlet was evaluated as a function of temperature and applied force. Figure 7 shows a 3D representation of the liquid–air interface in the simulations, and a plot of the simulated and measured results can be seen in Figure 8. Note that the simulated results are normalized. It was observed that the simulated results yielded three curves with increasing diameter for lower temperatures, while the experiments showed no significant differences between the spinning series at 290 and 300 °C. This can be attributed to little actual change in viscosity here, which can be caused by thermal degradation or the limited volume of the melting zone. The simulations cannot take this physical difference into account and will, therefore, deviate from the experimental results (Figure 8). Furthermore, as this effect takes place for the greatest spinning distances, the simulations cannot account for the whipping of the fiber either. 

**Figure 9:**
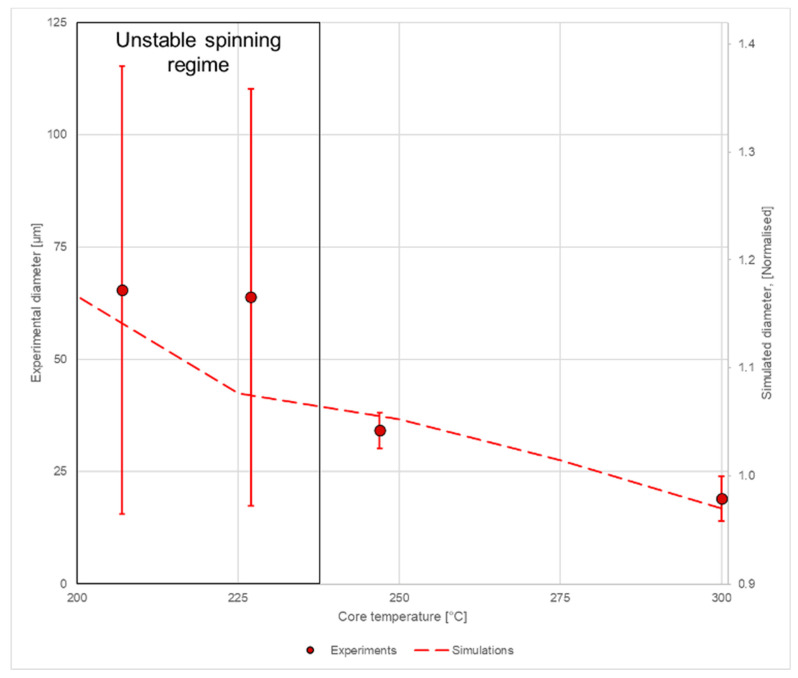
Simulated (line) and measured (dots with error bars) diameters for electrospun composite fibers. Note that the diameter for the simulations is normalized with respect to the smallest diameter. The area where the spinning process is unstable is marked in the gray box.

Figure 9 shows the relationship between the measured and simulated diameters for the composite fibers. While the low-airflow experiment (30 mL/s, 247 °C) yielded uniform fibers with a small diameter variation (34 ± 4 µm), the high-airflow experiments (40–50 mL/s, 227 and 207 °C) showed instabilities, yielding very high variance in the fiber diameter (64–65 ± 46–50 µm). The applied method was found to produce aerogel-containing fibers, and the modelling approach was able to reproduce the experimentally observed trend within the stable spinning region.

Furthermore, it can also be concluded that the primary obstacle in the electrospinning of composite fibers with pneumatic conveying is cooling by the transport gas. This type of issue might be avoided by applying an airflow around the spinning nozzle (e.g., [40]) where heated air is used to support the Taylor cone formation. Moreover, heated airflow might also be applied to the pneumatic gas to reduce the cooling effect. These types of additional studies could also form the basis for an update of the airflow and electrospinning simulation.

The simulation method can find applications as it describes the relationship between the electrospinning parameters, temperature, viscosity, spinning distance, and applied airflow. The experiments showed no whipping in the fiber-forming process due to the inversed setup and the relatively short spinning distance. Therefore, it can be assumed that a significant part of the diameter change occurs in the near field while the polymer is still viscous. This corresponds to the findings of Zhmayve et al. (2011) for crystallization during electrospinning [25]. 

In general, the applied ramping approach offers a way to simulate melt electrospinning in the temperature range close to the glass transition temperature where steep viscosity changes with temperature occur. The ramping approach offers a better computational robustness in comparison to the traditional William–Landel–Ferry model for thermoplastic viscosity [33]. Since the general temperature dependence of polymer viscosity is well-established, this technique can be applied to the development of new melt electrospinning equipment and techniques for a whole range of thermoplastic polymers. 

## 4. Conclusions

The increasing need for materials with new and improved properties calls for new production methods and the validation of these methods. Electrospinning of polymer/aerogel composites can yield novel thermal insulation materials and make them available for general use. 

The study showed that electrospun polymer/aerogel composite fibers can be produced solvent-free by the pneumatic transport of aerogel into a fiber core. The airflow of the pneumatic aerogel transport is the limiting factor of the aerogel loading into the fibers due to its cooling effect on the surrounding polymer. This leads to the fiber diameter increasing with increasing airflow due to faster cooling of the fiber core and the corresponding increase in the viscosity of the polymer melt. 

Finite element modeling of the melt electrospinning process was demonstrated. In order to accommodate the steep viscosity dependence on temperature in the simulations, a logistic function approach was applied to evaluate the viscosity of the polymer melt, coupled with a parametric ramping approach. A stable simulation of the composite electrospinning process was shown within the experimentally accessible range of parameters. 

## Figures and Tables

**Table 1:** Parameters applied to the polymer flow model.

Parameter	Value	Unit
$\mu_{melt}$	0.1	Pa·s
$\mu_{glass}$	0.1–10,000	Pa·s
Ramping factor between each simulation	10	-
a	0.001	-
$T_{glass}$	150	°C
$T_{melt}$	250	°C
$c_{p,polymer}$	0.1	W/m·°C

**Table 2:** Overview of fiber diameters for non-aerogel fibers from PET and cellulose acetate.

Material	Distance (cm)	Temperature (°C)	Diameter (µm)	STD.DEV (µm)
PET	7.4	280	29.5	8.3
PET	10.9		43.7	10.8
PET	17.4		56.8	10.8
PET	20.4		91.2	16.1
PET	10.1	290	25.3	6.8
PET	20.0		42.1	14.1
PET	20.9		36.4	8.3
PET	7.4	300	19.9	5.5
PET	10.1		29.0	7.0
PET	10.9		22.0	2.9
PET	16.9		50.3	9.3
PET	7.4	320	12.4	2.6
Cellulose Acetate	7.4	150	39.0	11.9
Cellulose Acetate	7.4	160	22.2	3.9

## Data Availability

Data will be made available on request.
